# Gendered traditional agroecological knowledge in agri-food systems: a systematic review

**DOI:** 10.1186/s13002-023-00576-6

**Published:** 2023-04-06

**Authors:** Ana G. Ramirez-Santos, Federica Ravera, Marta G. Rivera-Ferre, Mar Calvet-Nogués

**Affiliations:** 1 UNESCO Chair of Sustainability, Polytechnic University of Catalonia, C/ Colom, 1 – Edificio TR1, 08222, Terrassa, Barcelona, Spain; 2 Department of Geography, University of Girona, Pl. Ferrater i Mora, 1, 17004 Girona, Spain; 3 INGENIO (CSIC-Universitat Politècnica de València), Edifici 8E, 4ª planta, Universitat Politècnica de València, Camí de Vera, s/n, 46022 Valencia, Spain

**Keywords:** Adaptation, Agri-food system, Gendered knowledge, Gendered spaces, Traditional knowledge, Women

## Abstract

Traditional agroecological knowledge (i.e. TAeK) is gaining recognition for its potential contribution to climate change adaptation in food systems, ecosystems restoration and food insecurity. Despite the existing literature on Traditional Ecological Knowledge and its nexus with food security, how gender critically influences the distribution of such knowledge within agri-food systems has not yet been systematically analysed. In this regard, this systematic review attempts to answer four questions: 1) How does the literature on gender and TAeK in agri-food systems evolved temporally, geographically and in different agroecosystems? 2) How are gender and intersectionality mainly approached by such literature? 3) How do the articles address gendered dimensions in TAeK within the agri-food system activities? 4) What are the main drivers of change that influence TAeK and adaptive responses? The results show the gendered nature of TAeK in relation to food production, processing, and conservation activities, and how these activities are linked to tasks and activities, gender-specific knowledge, and spaces where gender discrimination is reproduced. The review also identifies elements that delimit and/or take part of the development of TAeK, such as gendered access to resources, gendered institutions, and the identification of the main drivers of change and impacts of TAeK erosion and biodiversity loss. These results are discussed in terms of power relations that interact with sociocultural norms and practices according to the specific geographical context and agroecosystem.

**Supplementary Information:**

The online version contains supplementary material available at 10.1186/s13002-023-00576-6.

## Introduction

Indigenous local knowledge (i.e. ILK), that is, the understandings, skills, and philosophies developed by societies with long histories of interaction with their natural environment [1, 2], plays a key role in the conservation and sustainable management of ecosystems, as well as in the adaptation to climate change and ecosystems’ resilience [3] For that reason, it has been widely acknowledged by scientists and international organizations like the Intergovernmental Platform Biodiversity Ecosystem Services (IPBES) [4] or the Intergovernmental Panel on Climate Change (IPCC) [5].

ILK is considered a body of knowledge and practices for the management of resources in a given context, not associated with any formal learning or training, but that contributes to conserving biodiversity and the sustainable use of natural resources in different ecosystems [6–8]. Indigenous knowledge (IK) is considered an important cultural component usually transmitted orally and by imitation [9, 10]. IK or folk knowledge refers to the unique knowledge confined to a particular culture or society, while local knowledge is more referred to context specific knowledge, for example, agroecological specific knowledge. ILK is often equated with traditional knowledge.

Adapted from these definitions, traditional agroecological knowledge (TAeK) is defined as a cumulative body of knowledge, traditions, practices, beliefs, institutions, and worldviews acquired through the direct dependence between cultural groups (Berkes [6]) and their agroecosystems and food systems, and generationally adapted and enriched over time [6, 11, 12].

TAeK integrates a deep understanding about the optimum management of agroecosystems functions in a culturally adapted way [12, 13] and contributes to food production, transformation and conservation, health enhancement and improved livelihoods and human welfare, including both biophysical aspects and cultural values [14, 15]. In the last few years, there has been a growing interest in understanding TAeK contributions to climate change adaptation, food security, and the restoration of ecosystems associated with food production. The IPCC noted that TAeK is absolutely necessary to build sustainable food systems capable to adapt to climate change and reduce greenhouse gas emissions [16]. In this line, a variety of empirical systematic reviews have: i) explored the importance of TAeK in the contribution of biodiversity conservation and environmental management, its quick erosion due different socio-economic and cultural factors, identifying different existing conservation initiatives to maintain this knowledge [17]; ii) shown how sustainable agricultural practices (such as integrated, soil, crop, landscape, water, and genetic management) can improve the resilience to climate change [18]; or iii) identified how agroecological practices contribute to the alleviation of community vulnerability [19]. Systematic reviews have also explored how ILK understandings (knowledge systems) are addressed in sustainable transformation research, and how the indigenous and transformation understandings are represented in the literature [18]. In addition, the connections between the set of agroecological practices affecting ecosystem services have been explored [20].

TAeK is gendered, with men and women holding different knowledge [19, 21]. Different reviews exist that analyse TAeK gender issues. Some have addressed the connections between gender and agrobiodiversity conservation, showing that gender relations determine and shape how women and men relate to, and interact with, environmental resources [19, 20, 22]. Women’s ILK has been discussed in relation to its crucial role in the market economy, to achieving the Sustainable Development Goals (i.e. SDGs), and its relevance to avoid serious consequences for the survival and development of local communities [23]. Culturally specific and dynamic relationships between gender and agroecological knowledge, considering age, ethnicity, cultural norms, the gender division of rights and responsibilities as critical elements that influence the acquisition and adaptation of local agroecological knowledge have been explored [24]. Under the lens of feminist political ecology (i.e. FPE)^1^, the identification of differential knowledge of men and women about natural resources and the experiences of inequality in accessing certain natural resources have been analysed [26].

Although there are studies that explore the gendered spheres of ILK, as the central role played by men and women in agrobiodiversity conservation, biodiversity management, anthropogenic landscapes, food resources, and the inequitable power structures that affect women’s access to resources, there is not a review that explore the importance of gender as a critical variable that influence TAeK in the whole agri-food system^2^ differentiated by type of agroecosystems.^3^ Since men and women have different, equal, or complementary TAeK about the production, transformation, and conservation of certain resources in specific agroecosystems, it is necessary to identify and make it visible the different daily experiences that men and women experiment and that critically affect the way in which this TAeK unfolds, transforms, and continues. In this respect, access to resources is considered as a critical element that influences the base for applying, adapting, modifying, transmitting, and maintaining TAeK and is integrated into the review analysis along with gendered tasks and activities, gendered knowledge, gendered crops, and gendered division of rights and responsibilities in spaces, which is referred such as gendered space. Addressing this gap of information is important to understand the gender-differentiated contributions and strategies required to conserve and use TAeK, as well as to address the gender inequalities and power structures affecting women and men’s knowledge in the agri-food system activities. This will allow to preserve TAeK and the agroecosystems on which women and men depend but also to better design adaptation policies that consider the differences in knowledge linked to gender.

This article aims at filling such gaps. To do this, four main questions are set: 1) How does the literature on gender and TAeK in agri-food systems evolved temporally, geographically and in different agroecosystems? 2) How are gender and intersectionality mainly approached by such literature? 3) How do the articles address gendered dimensions in TAeK within the agri-food system activities? 4) What are the main drivers of change that influence TAeK and adaptive responses?

## Materials and methods

### Literature review and data collection

**Fig. 1:**
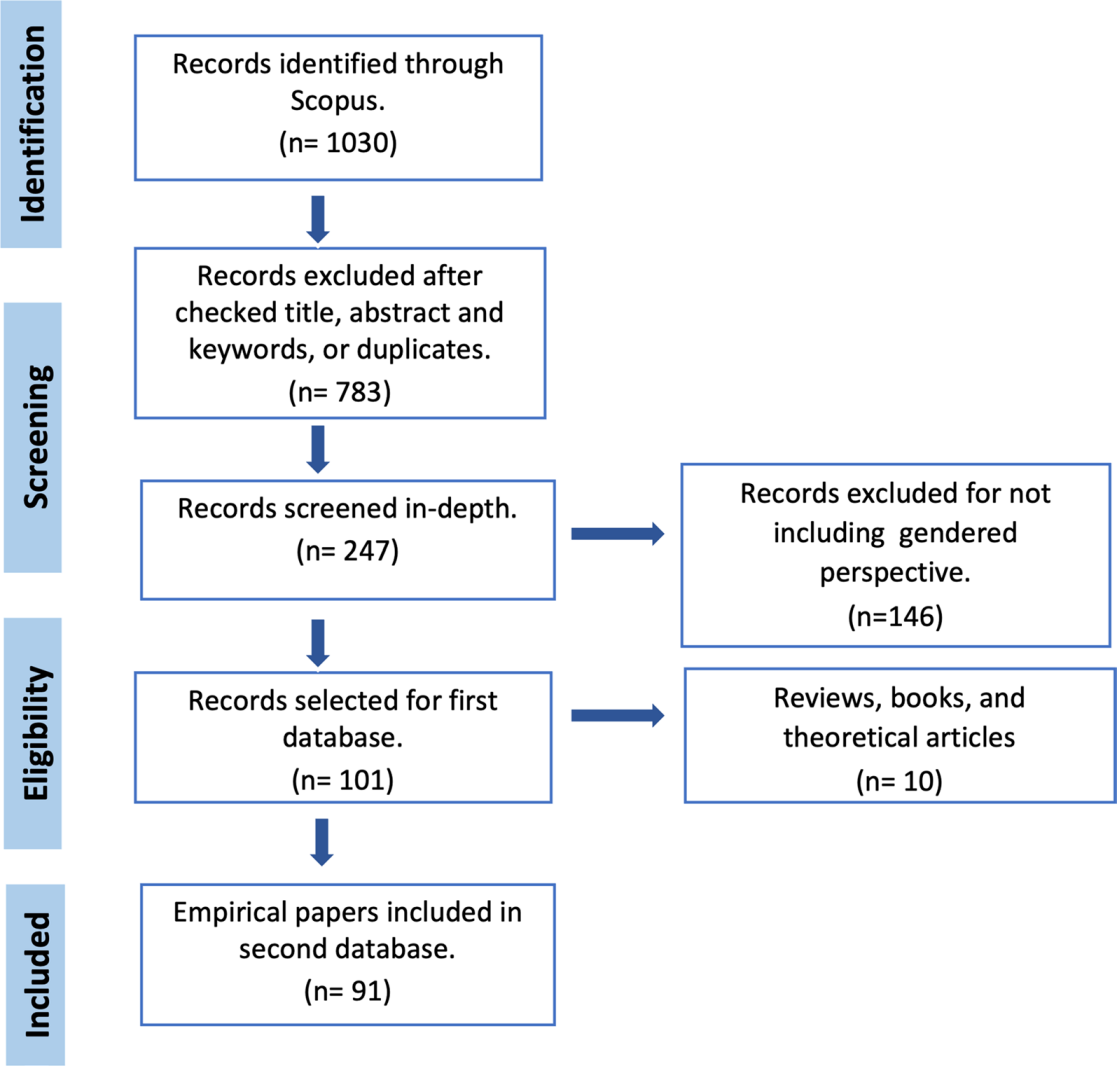
Flow diagram of the literature review process

We conducted a systematic review using the guidelines for Systematic Review in Conservation and Environmental Management [29]. The search of literature (updated in March 2020) was performed through the web database Scopus (https://www.scopus.com) with the double objective of 1) reviewing scientific literature on TAeK in agri-food systems and traditional agricultural knowledge and 2) specifically, reviewing it from a gender perspective. Three groups of root keywords were used around agri-food system, knowledge, and gender to make a simultaneous combination (e.g. "Agr*" AND "local knowledge" OR "indigenous knowledge" OR "ecological knowledge" AND "wom*" OR "fem*" OR "gender"). In a preliminary search, we obtained *N* = 1030 documents of which *N* = 247 were selected after screening title, abstract and keywords. Exclusion criteria included: documents that were not directly linked to agri-food systems; documents that did not incorporate gender as a theme. These papers were subsequently reviewed in depth, resulting in a further exclusion of *N* = 147, as they did not deeply include a gender analysis. We also excluded conference proceedings and brochures as well as papers related to fishing management leaving out the TAeK of aquatic resources and species since the focus of the analysis is on TAeK in agri-food systems. Mycology papers that did not study traditional management or did not focus on a case study were also excluded. However, internal watershed, such as streams and rivers, and freshwater wetlands management has been included because of its repercussions on agroecosystems. Of the 101 selected, 10 were books, theoretical and review papers, and were also excluded to avoid replication bias (see Fig. 1) (see Additional file 1).

## Results and discussion

### General overview of the literature in TAeK and gender

#### Temporal trends of publications

**Fig. 2:**
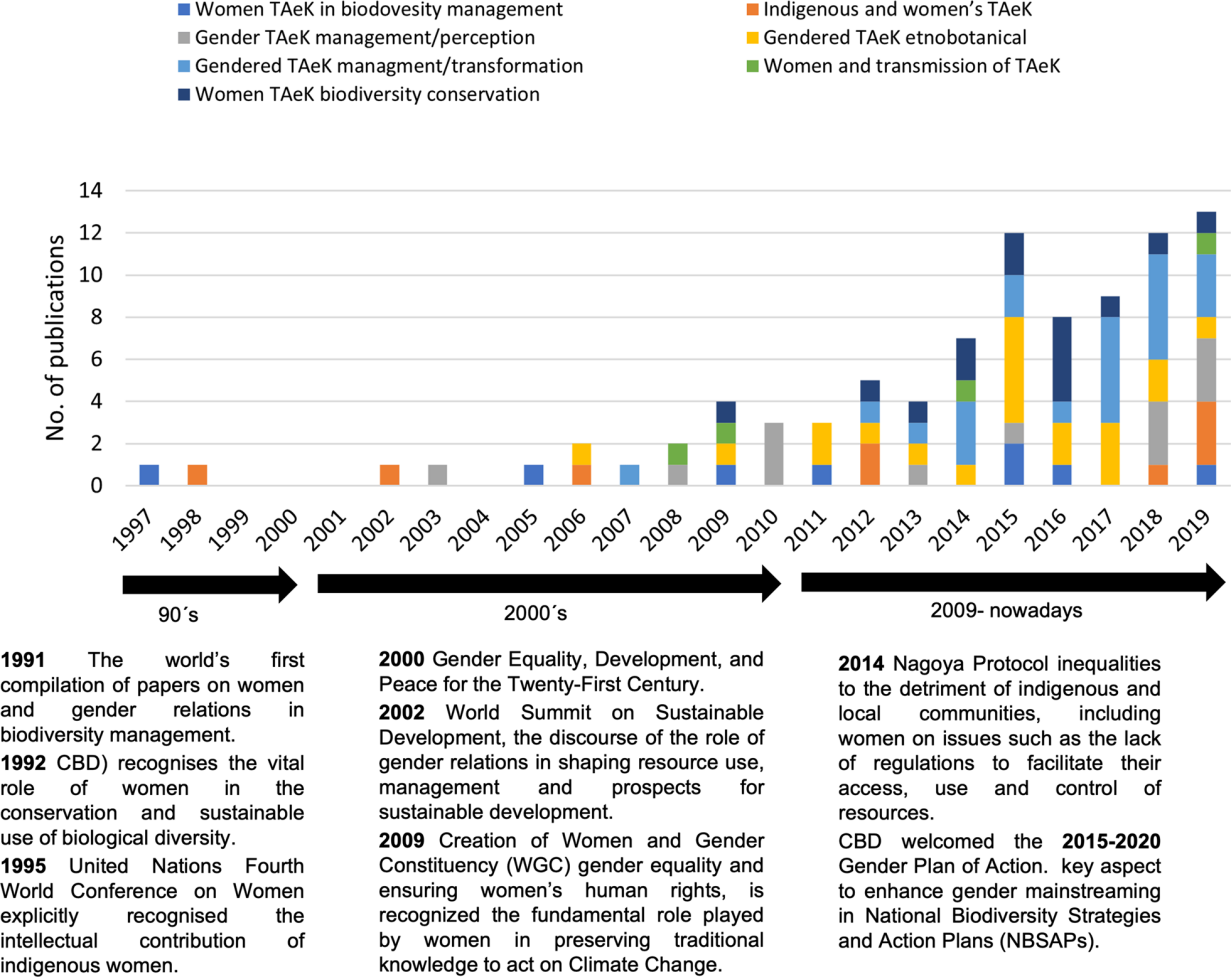
Temporal distribution of the 91 empirical papers (year of publication) analysed, the gendered TAeK topics addressed, and specific international events that could interact with the scientific production

In our database, the first empirical paper studying TAeK linked to the agri-food system from a gender perspective was published in 1997. The number of scientific publications modestly increased in 2009 (*N* = 4) and 2012 (*N* = 5), with the highest peak in 2015 (*N* = 12) and 2019 (*N* = 13) (Fig. 2).

In the 90s, there were very few studies on gender, most of them dealing with women’s issues rather than gender. The publications principally explored how indigenous groups and women’s environmental knowledge could potentially contribute to environmental management (*N* = 2). During the 2000s, gender was better integrated in the analysis, including studies on gendered knowledge and perceptions of management strategies (*N* = 6), gendered knowledge acquisition and transmission, also in relation to gender division of  labour (*N* = 5). From 2010 to present, the topics expanded to include understanding the role of institutions and other societal factors that influence the gendered dimensions of TAeK and its effects, such as the role of gendered participation and formal and informal institutions and networks in sustaining biological diversity (*N* = 3), or in maintaining and promoting agricultural genetic diversity resources to promote food security and food sovereignty (*N* = 11), the gendered division of labour in environmental management (*N* = 20); and the inclusion of a more intersectional approach, i.e. the intersection of gender with other sources of oppression, such as social status or age (*N* = 9).

#### Geographical distribution and agroecosystem types

**Fig. 3:**
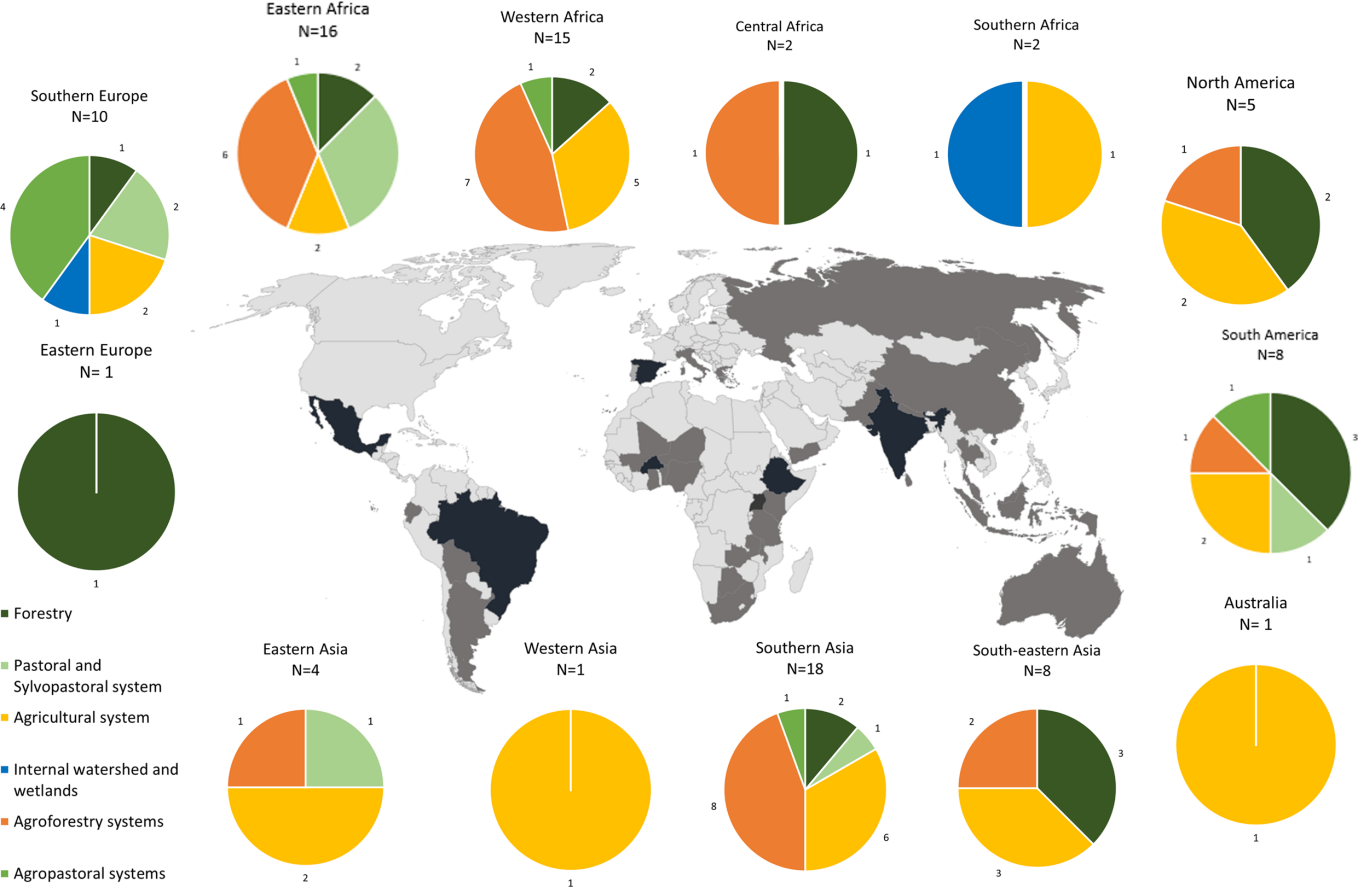
Geographical distribution. Number of papers published by country, and description of agroecosystem by geographical areas; deep black countries have more than 6 papers

The 91 empirical papers of our database were spread across 37 countries around the globe (Africa *N* = 35, America *N* = 13, Europe = 11; Asia = 31; Australia *N* = 1). Most of the papers were in the Global South (74%, *N* = 67), mainly India (*N* = 15), Burkina Faso (*N* = 6), Ethiopia (*N* = 6), Brazil (*N* = 5), and Mexico (*N* = 5); while only a few were in the Global North (26%, *N* = 24), especially in Spain (*N* = 7). Surprisingly, despite the rich endogenous cultures in Canada and the USA, we found no papers analysing gendered TAeK in that area using our search string (see Fig. 3).

Several studies identified specific cultural settings where TAeK is developed. African studies mainly referred to ethnic groups (*N* = 28), Asian to tribe communities (*N* = 9) followed by ethnic groups (*N* = 7), and Latin America studies mainly to Indigenous groups (*N* = 7). Also, the papers reported minority and marginalized groups, mainly in Asia (*N* = 9; *N* = 8) and Africa (*N* = 3, *N* = 6). European studies define TAeK as knowledge of rural communities (*N* = 9).

Concerning the agroecosystems analysed, agroforestry (*N* = 27) and agricultural system (*N* = 27) were the most documented.

Agroforestry was mostly documented in West Africa (*N* = 7), East Africa (*N* = 6), and South Asia (*N* = 6). The papers reported information on TAeK related to interactions between tree species, use of wild edible plants (WEPs) and integration of edible plants in traditional agroforestry design [30], and contribution of gathering practices in subsistence agriculture as food supplements [31]. TAeK related to gathering was the main practice described and allocated to women. Both men and women possess TAeK about identification of characteristics of plants (different uses of the different parts of the plant), period of harvesting, culinary, medicinal and ethnoveterinary uses. However, men and women tend to use native plants in different ways and show different degrees of knowledge in relation to age, the space, and geographic zone in which they operate or their gendered role [32, 33]. Yet, considering agricultural activity within agroforestry systems, women’s gathering knowledge was mainly related to subsistence farming and gathering food for family needs [34–39], while men were often responsible for gathering constructions and fodder resources in areas far from the household [31, 40].

Agricultural systems (*N* = 27) were mostly documented in South Asia (*N* = 6) and West Africa (*N* = 5). These studies mainly describe homegardens’ practices. Women and men show different forms of TAeK with specific reference to i) the conservation of genetic resources that involves knowledge of seed varieties, selection, preservation, and storage; e.g. in South Asia, women have accumulated immense knowledge of seed collection and seed preservation about a huge variety of vegetables and tubers [41]; in the same area, women of the Bar tribe are the major custodians in the conservation and management of rice seed for food production [42]; ii) cultivation methods, like in South Asia, where selection, conservation, and sowing of rice seeds is considered “men’s domain”, but women have extensive knowledge about rice varieties, seed selection techniques, cultivation methods of different rice seed species and pest control measures [41]; iii) indigenous crops and small-scale farming as an important component of food sovereignty; such as the case of South Africa where women grow indigenous varieties mainly in homegardens, making food available and avoiding grocery purchases [43]; in particular in West Africa, the importance of small-scale vegetable production in the family diet and generating household income is highlighted, e.g. Amaranth species are the most cultivated, as they are consumed during all seasons and used for many dishes in the local kitchens, and women play and important role in their commercialization [44]; iv) irrigation methods, especially in drylands; as in South Asia where in order to adapt to climate change, men have gradually improved their irrigation infrastructure through irrigation canals, reservoirs, and water diversion systems to maintain agricultural production [45, 46].

Pastoral (*N* = 10) and agropastoral systems (*N* = 8) from a food system and gender perspective were mainly described in Africa and Europe. In East Africa (*N* = 6), women play a vital role within the pastoral system, even if they have been referred to as the “hidden hands” in spite of they are primarily responsible for taking care of smaller, younger, and sick animals around the home, and they have TAeK of milking, milk processing and marketing [47]; however, in West Africa men tend to be more knowledgeable about livestock in many traditional societies [48]. In Europe (*N* = 6), the literature addressed men and women TAeK related to the transformation and food processing of meat (*N* = 3) and dairy products (*N* = 3) [49], and one paper described men and women TAeK related to pastoralist practice and transhumance, where very few women are fully involved in transhumance because most of the daughters of transhumance herders migrate or are employed in other activities (*N* = 1) [50]. In Asia (*N* = 2), for instance, studies on gender roles in livestock have indicated that feeding, milking, cleaning, caring for the animals, administering medication, are mainly carried out by women [51], and the relation of informal social networks, namely friendship and the practice of migration, in the distribution of knowledge about soils, ethnoveterinary and sheep breeds among male and female shepherds [52].

Forestry (*N* = 16) was mainly described in Asia (*N* = 5), Africa (*N* = 5), and America (*N* = 4); in South Est Asia (*N* = 3) papers noted gender-based differentiation in gathering of WEPs [32]; in West Africa (*N* = 1) men and women’s knowledge uses of fruit tree species [53]. In South America (*N* = 2), gathering practices within forest showed gendered TAeK related to men and women’s ethnobotanical knowledge and the use and management of plant species for food and medicinal purposes [54], and medicinal knowledge contributions to health sovereignty [55].

Globally, the least addressed ecosystem was internal watershed/freshwater wetlands (*N* = 2), with one case in each of the geographical areas of South Africa and Europe. In South Africa (*N* = 1), the paper addressed men and women’s knowledge related to the flood recession farming systems in communities residing along river systems and describes the knowledge about risks of floodings [56]. In the case of Europe, this knowledge is greater among men, probably due to the “masculinization” process that has taken place in rural communities [57].

### Approaches to gender and intersectionality adopted

Gender is addressed mainly in the methods (*N* = 23) operating as a variable of data analysis used as a component that helps in the identification of the knowledge distribution among genders. Gender is presented in the discussion addressing gendered division of labour within the agri-food system activities of production, processing and conservation (*N* = 65), the distribution of TAeK among women and men in specific society, community and agroecosystem (*N* = 15), gendered perceptions related to climate change effects (*N* = 5), gendered perceptions of the natural environment and food resources (*N* = 3), gendered perception of vulnerability related to climate change factors (*N* = 1) [47], and also the concept of gendered blind since in the analysis the gender was not a significant cultural attribute for knowledge (*N* = 1) [36].

The results addressed mainly gender variable distribution and different levels of TAeK between men and women combining other elements such as demographic variables, i.e. age and level of education, and agroecosystem site characteristics, i.e. altitude and climate (*N* = 21).

A few papers addressed specific gender approaches in the literature, mainly FPE (*N* = 1) and intersectionality (*N* = 8). From these perspectives, the intersection of gender, ethnicity, and age has been referred to as elements that can significantly shape the TAeK body in specific ecosystems [48, 58, 59] and have a direct impact on the decline and disappearance of TAeK [60]. In South Africa, the intersection of race and indigenous categories, in addition to gender, deals with the challenging experiences of racialized indigenous women to continue with cultivation practices to achieve food sovereignty [43]. In South Asia it is analysed how development initiatives have failed to integrate and enhance women’s knowledge related to agriculture and improved food security, since the construction and transformation of that TAeK is largely dependent on government and community that are still maintained in patriarchal power structures [41]. Other authors (*N* = 3) consider how the intersection of gender and class shapes inequalities and negative impact on women’s access, management, and control over resources [26, 41, 61], or on women’s unequal access to knowledge about land rights, resource tenure, and external technologies and practices that emanate from formal institutions [46].

### Gendered TAeK in agri-food system activities

This section presents an analysis of gendered access to resources and gendered institutions, as elements that can potentially affect the development, acquisition, permanence, and continuity of TAeK. Subsequently, it shows how the different gendered dimensions of TAeK within the agri-food system activities are addressed, considering geographical context and agroecosystem type.

#### Gendered access to resources

Articles addressed gendered issues related to the access to land, seeds, and forests as potential barriers to the use of TAeK. Regarding *access to land* (*N* = 13), the literature addresses land tenure which is governed by customary laws based on an intergenerational transfer of land in patrilineal societies where all inheritance rights go to the male (*N* = 4), who also represents the family in its external relations over the use of natural resources in the communities or villages [60, 62, 63]. In other cases (*N* = 5), women can only access land through secondary channels as their family membership or marriage or otherwise, their control over the resource base is negligible or nil [35, 62, 64, 65]. In West Africa, older male heads allocate individual fields and communal family fields, assigning the largest fields with higher levels of soil fertility to male members [64]. In South Asia, one case described gender differences of maintaining land rights, and gendered exclusion due to lack of access to the social networks and institutions that allocate land resources. Since gender equity is not promoted in the formalization of individual land titles, women consider land titles as unfair because it is often given to men [41].

Gendered tenure regimes have different implications. In forestry systems, it is affecting management strategies, women knowledge, access, and control of forest and trees’ resources in Africa (*N* = 2) [62, 66], while in Asia (*N* = 2), the limitation of women in decision-making and participation in forestry results in their limited access to forest resources [41, 51]. The gender division of labour or gender roles that privilege men in the *access to land* give them more access and control over joint family resources, e.g. land and water, while women are exposed to a double workload of both reproductive responsibilities and on-farm activities, which limits their capacity to generate relationships, create networks, make independent decisions about their resources or gained/acquired knowledge of land allocation process [43, 46, 51, 64, 66, 67].

Although generally seed collection, preservation, and knowledge associated with them are largely the domain of women [41, 43, 68, 69], in South Asia (*N* = 3) it is described that resource management for agriculture and agrobiodiversity knowledge follows well-defined gender roles that privilege men; even though Kurichya women have extensive knowledge of rice cultivation, they cannot use it for actively cultivating rice on their own, as they have no access to traditional rice seeds and land in the rainy season [41]; in West Africa among the factors that were identified by young and elderly women, access to household granaries was the greatest worry since their husbands denied them access to the household granaries, as crop yields were decreasing due to climate variability [61]; another particular case in West Africa addressed men’s and women’s access to seeds through seed banks; the analysis was done from FPE and found over the years the banks disappeared, the main reason was that women in the Upper West Region of Ghana were systematically marginalized despite they play a key role in agriculture and seed selection[70]. As a traditionally male-dominated society, from their perspective women were not suited to these responsibilities [71].

In the *access to domestic granaries* (*N* = 2, in Asia and Africa), strong norms of patriarchy and socially constructed relations of gender and property rights restrict women to take food. Although women help to conserve and produce seeds, men allocate the quantities of grain for daily consumption, and women’s greatest concern was that their husbands would deny them access at times when crop yields decrease in the face of climate variability [41, 61].

Regarding the *access to water* (*N* = 2), papers in East Africa and South Asia showed the management of water resources for agriculture follows clear-cut gendered roles that privilege men[46]. Irrigation water is generally decided by men, who influence associations responsible for infrastructure and determine allocation schedules, without considering women’s specific concerns [51].

#### Gendered institutions

Informal (*N* = 11) and formal (*N* = 1) gendered institutions are considered in the literature. The informal networks are considered a crucial element for the continuity and transmission of the knowledge related to agrobiodiversity conservation and biodiversity management where women interact among the community to transmit and continue with the knowledge (*N* = 5). Another type of informal networks of women is created through socialization within the community and is key elements of supporting women’s activities within the different agroecosystem and, additionally, reproductive work, since these networks allow them to carry out reproductive activities and tasks within a network of support and mutual help (*N* = 6). A formal institution of women farmers’ group that has been promoted by the World Bank in India is presented, and this is recognized as a development action that aims to promote sustainable development initiatives within the women farmers’ group to generate a positive impact on agrobiodiversity, but they have had little success since patriarchal power structures concern the decision-making processes in the women’s group [41].

#### Gendered tasks and activities

**Fig. 4:**
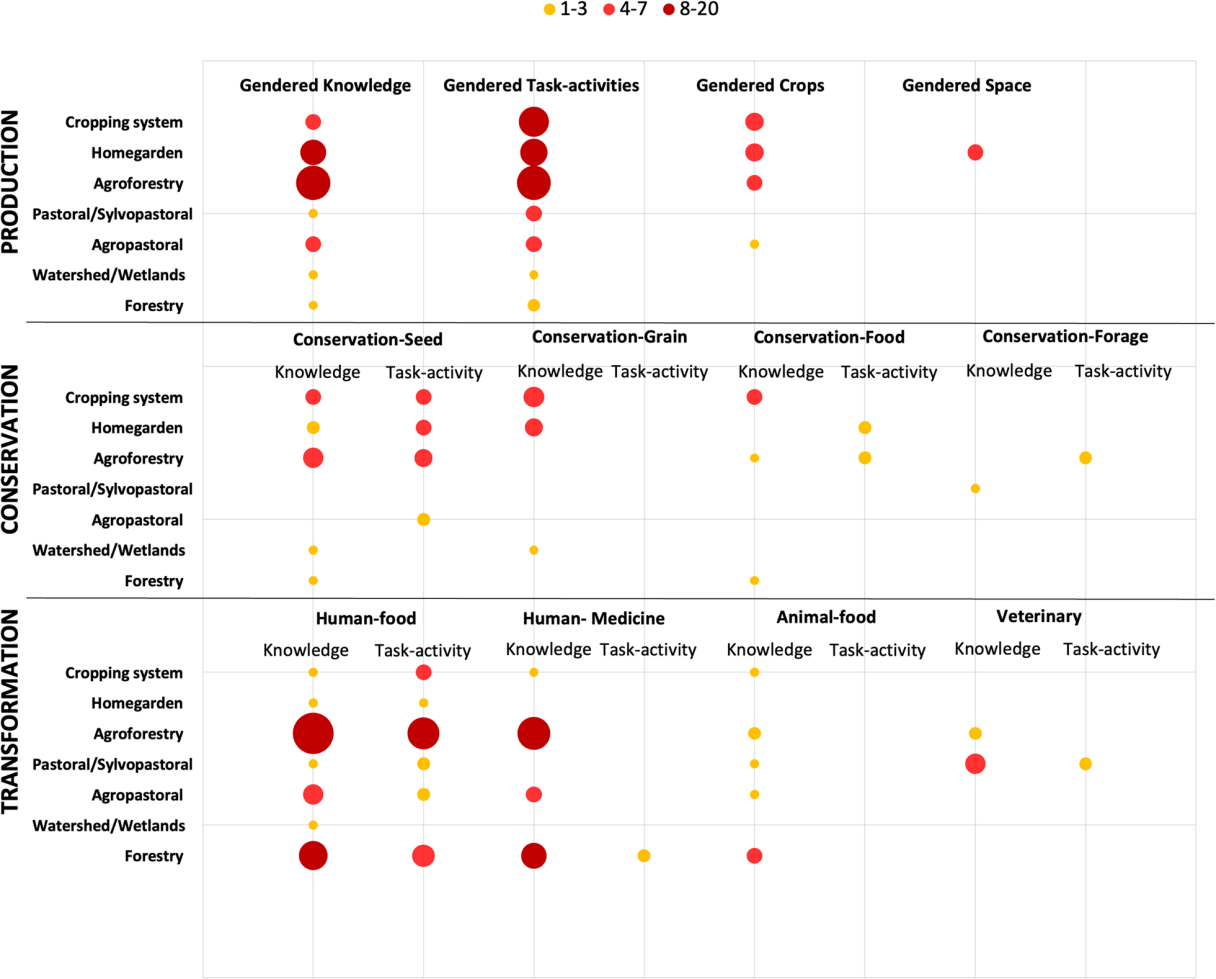
Number of papers addressing TAeK and gendered dimension in production, conservation, and transformation links in different agroecosystems

Aspects such as gendered tasks and activities, gendered knowledge, gendered crops, and gendered space are detailed below (see Fig. 4).

##### Production.

Gendered tasks and activities in production were widely described in *agroforestry systems* (*N* = 13). In some literature in South Asia (*N* = 7) and West Africa (*N* = 2) tasks and activities include gathering of forest products for food, fuel, fodder, medicine, and small-scale trade, which are generally carried out by women. One paper in South Asia highlights the existence of asymmetrical pressure on women and/or elders, due to women’s roles in managing resources, fuel, water, and medicinal plants, which requires walking increasing distances and leaves less time to care for themselves, their children and to participate in education and village governance [72]. In West Africa men contribute to collect fuel, animal products, and the extraction of structural fibre for construction or sale [73]. Gendered division of labour in agroforestry influences men’s and women’s relationship with local woodlands, where women are mainly involved in collection and transformation processes of non-timber forest products for sale [62].

The gendered tasks and activities that women and men perform in the agricultural part of agroforestry concern land preparation. In one case in South Asia, the chemical spraying and fertilizer application are equal, but women perform the irrigation [51]. In South-East Asia, men are more active than women in land clearing, weeding, cleaning, pruning, and burning considered heavy tasks [74].

In *Cropping system (MINUSCULE)* in West Africa (*N* = 5, South Africa (*N* = 3), and East Asia (*N* = 2) the gender division of labour in the allocation of activities shows that women not only work as the unpaid family worker in agriculture and other occupations but also hold care-giving responsibilities for children and elderly people. In South Africa the ‘traditional’ Zulu culture, women’s task relates to cultivate, while men work in the cities or tend cattle [43]. In East Asia, women’s task in household and farming, men are also engaged in farming and off-farm work for wages, with a greater decision-making power in the production and domestic area [45].

In *homegarden systems*, several papers (*N* = 7) in Africa, Asia, and Europe have noted that women are responsible for vegetable production and (*N* = 2) additionally highlight that these activities are performed to grow crops to supplement purchased food [41, 43, 68, 75–77]. One paper in Africa describes that women perform more tasks in homegardens and are more involved in weeding, irrigation, and planting, while men’s activity is limited to fencing the area [76]. In Central America, the task of building earth ovens is carried out by men, and preparing food is performed by women; the activities associated with the milpa only in some cases are an exclusively male task, but usually involve the whole family [78].

In *forestry system (MINUSCULE)* in South Asia, management is usually in the hands of men, and they select the cultivation site, while women accompany them in harvesting, burning, and clearing [79]; in Central Africa, women activities are involved in harvesting mushrooms and are the main holders of cultural aspects related to fungi [80].

In *pastoral and agropastoral systems* (*N* = 4), gender-based division of labour typically assigns livestock-raising practices to men. In Europe, very few women are fully involved in transhumance, mostly because of generational renewal problems and agrarian masculinization [50, 81]. In Africa and Asia, women’s activities are often overlooked, but they play a vital role in pastoral production, taking care of the livestock and sick animals, feeding, milking, milk processing, and marketing when men migrate for long periods in search of pasture or markets [47, 51, 82].

##### Conservation.

Gendered tasks and activities in seed conservation were described in *agroforestry* (*N* = 4), *cropping* (*N* = 3), *homegarden* (*N* = 3), and *agropastoral systems* (*N* = 2). In South Asia (*N* = 3), women’s tasks related to seed collection allow them to have a huge variety of grains, vegetables, and tubers contributing to the conservation of agrobiodiversity [41, 42]. In South Africa, due to the cultural norms that delegate to women the responsibility of maintaining the household food supply, they have primary responsibility for seed collection and storage activities [43, 60]. In Australia, knowledge of edible seeds, including their ecology and mythology, is extensive, as is the development of specific tasks such as singing songs to collect, preserve, and process them, as it is part of the contemporary cultural identity [83]. Concerning forage conservation task and activities described in agroforestry system (*N* = 2), in South Asia women collect fodder from forest which along with other household responsibility [84]; in West Africa men are in charge of the activities related to fodder banks for dairy farms [85].

##### Transformation.

The tasks and activities for human food preparation were usually described as directly assigned to, or performed by, an individual in the community based on their cultural setting. However, gendered roles determine women as responsible for food/nutrition and food preparation in most places [34–36, 38, 39]. In East Africa, cooking is considered as women’s tasks and females were responsible for cooking and transfer of knowledge to younger members, while males spend most of the time grazing animals and hunting [31, 36]. In agricultural systems in Europe, artisanal food processing allows women to combine family care and this activity that also provides some income [86].

Task and activities linked to *human medicine* were marginally addressed. In *forestry systems* (*N* = 2), medicinal uses of resources were more important for women as compared to men [87]. Also, in Africa women activities inside the kitchen were connected to sell food and medicinal products [53]. *Veterinary* activity in *pastoral* and *silvopastoral systems* in South-East Asia is related to men, who are the main practitioners of ethnoveterinary tasks, as local tradition limits females to be involved in outdoor activities [88]. No elements related to tasks and activities in grain conservation and animal food were found.

#### Gendered knowledge

##### Production.

Gendered knowledge in production was mainly described in *agroforestry systems* (*N* = 9), in South Asia (*N* = 6) and West Africa (*N* = 3).

In South Asia (*N* = 2), articles addressed that women have a rich and diversified knowledge in livestock care and agriculture-based livelihoods [84, 89].  Crop associations prevent the failure of crop varieties under adverse conditions and pest-disease pressure. Although this is an important practice to ensure family’s food and nutrition security, women’s knowledge remains less important, and men dominate agricultural development [75].

Women were reported to be more knowledgeable than men about insect pests, disease infestations in trees, leaf collection dates, storage methods, and compost preparation [90]; they are aware of traditional practices, such as burning degraded hills to clear plots to grow leafy vegetables [91], controlled burning and logging of closely clustered trees to receive nutrient inputs and protected from squirrels and forest fires [73]. In South Asia, (*N* = 1) it is explained elderly women’s knowledge of vertical distribution of plant species across the community forest and homegardens, and knowledge of biometeorology (the effect of weather on plants and animals), needed to predict weather patterns and seasons [91]. In West Africa (*N* = 1) men’s and women’s differences in knowledge are pronounced, and many of these differences stem from a gendered division of household labour that extends to household agriculture and agroforestry; gendered engagement in local agroecosystems, related to the tasks and activities on which women and men centre, engendering different agroecological knowledge related to soil fertility and vegetation[62].

In *homegardens systems* (*N* = 5), articles located in South Asia (*N* = 2), West Asia (*N* = 1), and South Africa (*N* = 1) described women’s knowledge with WEPs, as they represent the main, and in some cases the only, source of food between field harvests or during crop failures [43]. In South Asia (*N* = 1), women knowledge was associated with farm management, seed selection, and genetic preservation [92]. In West Asia, much of the work related to homegardening is conducted by women, and they have the most knowledge and make most of the decisions regarding this space. From sowing to harvesting, there is relatively little involvement of men, but more for hard physical work such as building fences or digging wells. The participation of the whole family increases when the homegarden is a source of money [68].

##### Conservation.

We divide the conservation activity into seed, grain, food, and forage conservation. For each of them, we describe the gendered knowledge, tasks, and activities in different geographical contexts and agroecosystems (see Fig. 4).

Regarding *gendered knowledge* related to seed conservation in *agroforestry* (*N* = 5), *cropping* (*N* = 3), and *homegarden* (*N* = 2)*,* in south Asia, the literature highlights women knowledge and experience in maintaining agricultural genetic diversity, as an important element to enhance food security and adapting to climatic variability [43, 74, 75, 84, 90]. In addition, elder community members and women were noted as the real custodians of knowledge of traditional crop varieties, traditional seed management, classifications of seeds, exchange systems, and sociocultural institutions that support the continuation of conservation practices [91].

Women present knowledge in food conservation in South-East Asia, East Asia, West Africa and East Europe, the knowledge is related to storage [93] as solar drying mushrooms [94] and sun-drying techniques [33], The sun-drying are used to preserve different leafy plants and foods, so that after drying they can be used fresh, boiled, or fried [95]. In West Africa, smoked foods, use of sacks for storage, sun-drying techniques are considered optimal for the survival of households during food scarcity and family health [67]. Forage conservation knowledge was mentioned in East Africa (*N* = 1) knowledge on sustainable utilization of fodder species resources for grazers was presented by both men and women [96].

##### Transformation.

We divide transformation activity into human and animal food and medicine. For each of them we describe the gendered knowledge, tasks and activities in different geographical context and agroecosystems (see Fig. 4).

*Gendered knowledge* of human food transformation was widely addressed in *agroforestry* (*N* = 20) and *forestry systems* (*N* = 10). Women and men knowledge related to processing of WEP’s used as nutritional supplements in *N* = 12. Women’s culinary skills in using forest-based ethnobotanicals in traditional foods in *N* = 8 papers [63, 97, 98]. In Livestock systems in Europe (*N* = 2), women, elderly mothers in some cases, are the ones holding the goat cheese production knowledge, and small handmade ruminant-derived products as meat products, cheeses, dairy produce [49, 82].

Gendered knowledge of transformation in *human medicine* was overall identified in *agroforestry* (*N* = 13) and *forestry systems* (*N* = 8). A good part of the knowledge of medicinal plants was related to women’s role of caretaking to family [38, 39, 91, 98]. Here, there is not a clear-cut trend. For instance, in Europe [99] and West Africa [35] men hold more knowledge than women, which reflects the central role played by the cultural context, which defines the spaces in which each gender relates and connects with the natural environment. For example, sometimes sociocultural elements, taboos, prohibitions, or magical beliefs assume that men have certain power to use certain species for medicinal treatments [54, 100, 101].

Gendered knowledge to animal food was mainly addressed in *forestry* (*N* = 3), *agroforestry* (*N* = 2) and livestock systems (*N* = 3). Here, the literature revised showed that males have better ability and knowledge than women to identify forage and fodder species [39, 40, 48, 79, 87, 88, 102].

Gendered knowledge related to veterinary was mainly described in *agroforestry* (*N* = 2) and *pastoral/silvopastoral* systems (*N* = 5). Papers described that women hold less knowledge of ethnoveterinary medicinal native plants and WEPs than men [88, 103, 104]. For example, in East Africa, gender distributions of medicinal plant knowledge showed most of the traditional animal healers are males, this could be related to the local tradition of restricting these practices mainly to men, while women are not allowed to participate in outdoor activities, but stay at home taking care of babies and performing domestic activities, so their veterinary knowledge was limited to the use of plants found in domestic environments [88], as a result, women tend to know medicinal practices related to animals that are closer to the household [88, 103, 104].

#### Gendered crops and gendered space

The existence of *gendered crops* was described in *cropping systems* (*N* = 3) [41, 44, 76]; and *homegardens* in South Asia, West Africa, and East Africa (*N* = 3)[30, 62, 93], with men more involved in cash crops such as coffee or rice seeds, and women in vegetables and subsistence crops.

Women’s space (*N* = 2) was analysed by only two papers that identified *homegarden systems* as women’s area in South Asia [41]and South Africa; here they referred to small-scale vegetable production ([21, 43].

### Drivers of change of TAeK: impacts and adaptation strategies

#### Drivers of change of TAeK and main impacts

The main drivers of change of TAeK detected are socio-economic and cultural changes (*N* = 36), environmental changes (*N* = 14), and agri-food policies implementations (*N* = 8). The main impacts of the drivers were related to knowledge erosion (*N* = 21) and biodiversity loss (*N* = 20) (see Table 1).

**Table 1:** Papers identified that relate to TAeK’s drivers of change and impacts

Drivers of change	Subjects	Main Impacts
Socio-economic and cultural change	*Socio-political interventions* to foster social development, and knowledge holders’ integration into market economies [93, 96, 105]	*Knowledge erosion* Due to the technological implementation in response to market forces [12, 50, 57, 69] *Biodiversity loss* Due to the ever-growing human population [74, 106] Due to mining and logging [93]
	*New generations less likely to take part* in traditional practices with a clear intergenerational gap in knowledge transmission [31, 64, 96, 107]	*Knowledge erosion* Due to the modern development and fast changing social dynamics decreasing the cohesiveness of the sociocultural institutions [42, 75, 90] Due to the decreasing transmission from elders to younger people [63, 87, 94, 108]
	*Local traditions compete with modern ways of life* [94]	*Knowledge erosion* Due to the acculturation and loss of local languages [38, 95, 106, 107, 109] Due to social, cultural changes caused by the increase in tourism, improved roadways, expanding urban centres [110]
	*Fast changing sociocultural values and acculturation*, *and modernity* negatively impacting the management and conservation of biological resources [91, 111, 112]	*Biodiversity loss* Closely linked to the erosion of cultural diversity [57] Due to loss of social learning institutions [113]
	*Migration* for work, a modern lifestyle education, or climate change threaten food security [51, 72, 94, 114, 115]	*Knowledge erosion* Due to outmigration [57] *Biodiversity loss* Migration for outside work [65]
Environmental changes	Due to *climate change* (e.g. drought and floods, climate variability) [84, 116, 117]	*Biodiversity loss* Due to the impact of climate change [30, 44, 48, 56, 103, 104]
Agri-food policies	Oriented to *mechanization, modernization*, large extensions of monocultures and *the integration of external elements*, mostly subsidized, as fertilizers, seeds, and insecticides [51, 92, 118]	*Knowledge erosion* Due to the impact of the Green Revolution program on farming activities [92] *Biodiversity loss* Due to the energy-intensive agriculture [75] Due to overharvesting [119]
	*Increased support for large landowners* and the abandonment of smallholder development[120]	

Among some of the drivers found, we can mention those related to socio-economic and cultural changes, where the papers mention socio-political intervention such as new infrastructure, and knowledge holders’ integration into market economies [93, 96, 105], local traditions that compete with modern ways of life [56], migration [114], and one paper in Europe addressed the masculinization in rural communities, with women leaving agriculture to a greater extent than men, for the acquisition of higher educational qualifications as a mean to break with the patriarchal European agrarian context [115]. In relation to agri-food policies, one paper in South Asia addressed the introduction of new rice varieties, inorganic fertilizers, synthetic pesticides, and hand tractors, as factor of drastic change for the jobs of both men and women, or employment opportunities lost directly for many rural women; consequently, the local experiences and knowledge of many women farmers have been eroded or lost [92]. Related to environmental changes, one paper located in South Asia identified that women are often the most affected by reduced food and nutrition security due to their limited access to resources and the responsibility attributed to them for family reproduction [84].

#### Main adaptation strategies

Different adaptation strategies adopted by women (*N* = 4), men (*N* = 1), and no gender specified (*N* = 5) to cope with biodiversity loss and knowledge erosion were identified (see Table 2).

**Table 2:** Number of papers that identify different adaptation strategies

Adopted by women	Biodiversity loss	Use crop residues, weeds, ashes and manure as fertilizers, shrubs are planted near the house to reduce soil depletion and are used as living fences to protect from predators [76] Gathering practices (i.e. wild plants) [99] Decision-making including environmental practices and livelihood strategies [121]
	Knowledge erosion	Informal institutions to transfer knowledge and practices from one generation to another [74] Informal networks developed by women farmers linking other women to share knowledge[43]
Adopted by men	Biodiversity loss	Adaptive strategies of mobility, diversification, selection, communal pooling, and forecasting allows adaptation to climate variability [50]
	Knowledge erosion	–
No gender specified	Biodiversity loss	The role of companion trees in generating favourable microclimatic conditions as a strategy for climate change adaptation and mitigation [30] Traditional soil and water conservation techniques for semi-arid and Mediterranean environments [57] Traditional agroforestry practices saving multipurpose trees to promote soil moisture resilience, impact mulching, and provide microhabitats [103] Famers’ agroecological knowledge and cropping strategies [62]
	Knowledge erosion	The social group as a community incorporate new practices/technologies, generate hybrid knowledge suggesting local capacity for socio-ecological resilience [69] Initiatives that seek the scaling-up and scaling-out of agroecology through the digital common’s movement [12]

The strategies mainly adopted by women (*N* = 4) to address biodiversity loss are related to reducing soil depletion and protecting crops from predators [76]; and wild plants gathering practices considered as an adaptation strategy in periods of food scarcity [99]. To cope with knowledge erosion, informal institutions to transfer knowledge and practices (i.e. seed conservation practices, conserving and sustaining local biodiversity) from one generation to another [74] and informal networks to share knowledge and improve their food security [44] have been described. Only one strategy adopted by men (*N* = 1) was identified to face biodiversity loss in the agropastoral system in Europe, and this case can be explained by the fact that very few women are fully involved in transhumance because in this context they emigrate to study or find employment [50]. Adaptive strategies without specifying gender (*N* = 5) described mainly techniques to cope biodiversity loss, such as the use of companion trees in agroforestry systems as a climate change adaptation and mitigation strategy [30]; traditional techniques for soil and water conservation [57], and traditional agroforestry practices to promote soil moisture [103]. To address knowledge erosion were presented initiatives that seek the scaling-up and scaling-out of agroecology through the digital common’s movement [122], and the incorporation of new practices/technologies, generating a hybrid knowledge that suggests the local capacity for socio-ecological resilience [69].

## Final remarks and future research

This systematic review has provided an opportunity to overview the gendered nature of TAeK in relation to agri-food activities of production, transformation, and conservation, and how these activities are linked with specific gendered task and activities, gendered knowledge, gendered spaces where gender discrimination is reproduced linked to power relations that interact with sociocultural norms and practices.

The TAeK that men and women own, create, transform, delegate, or transmit within a specific geographical area and a particular type of agroecosystem is directly linked to collectively created cultural aspects, norms, rules, and laws that are not static, as these can either endure or be transformed along with the development of specific social dynamics, or drivers of change such as environmental changes, some of them related to climate change, or socio-economic, cultural aspects, and food policies that are intertwined with power structures and relations that directly affect the construction and erosion of knowledge and biodiversity loss [123].

Since this systematic review analyses gender as a fundamental element that influences TAeK, access to resources of women and men to certain resources is identified as critically influencing the construction, adaptation, as well as modifications and ways of transmission and maintenance of this body of knowledge; also, in the capacity of men and women to ensuring food resources and life-sustaining resources daily. Access to land is one of the most identified issues, showing that gender division of labour and gender roles privilege men in access to land, as well as customary laws in patrilineal societies in which all land inheritance rights go to men. In terms of access to seeds, women are mostly considered to be the guardians and linked to the conservation of genetic resources, but in some cultures the power structure based on patriarchal logic favours the male figure, and women have little or no access to resources. For example, in the cases of West Africa and South Asia, women do not have access to traditional seeds and land in the rainy season, and when crop yields decrease due to climate variability, access to granaries and food is limited by men. In addition to this privilege of men with greater access to and control over joint family resources, in some cultures, specific seeds and crops (i.e. such as coffee) are considered men’ crops.

Knowledge is also associated with the tasks and activities performed by men and women, and this review delves into the fact that in different agroecosystems there is a gendered division of household labour that extends to tasks and activities within production, conservation, and processing activities. On the one hand, based on patriarchal logic, gender roles have been established that support the idea that women are responsible for reproductive work, care, and feeding the family, and in some cases it is found that women perform tasks and activities to ensure the satisfaction of these needs, as in the agroforestry system where they are the ones who must walk long distances in search of resources to meet the nutritional and medicinal needs of the family. Another example is the situation of women in agricultural systems, which is aggravated by the double workload, which implies carrying out domestic chores, but also the work of producing, conserving, and transforming food. On the other hand, patriarchal structure through the division of labour frequently associates men roles with activities outside the domestic area such as the transhumance where very few women are involved. This analysis also reveals that the construction of gender roles in specific cultures and agroecosystems is related to the acquisition, creation, and transmission of TAeK. In most cases within agroforestry and forestry systems, it is women who have greater TAeK associated with specific activities such as resource collection and transformation of these for the family food supply, and in the case of men this knowledge is associated with the collection of fuel, animal products, and construction materials. In addition, cases were found where there are different degrees of knowledge related to gathering in men and women in relation to age, space, and geography. In addition, greater TAeK related to transformation and processing of human food and medicine was found in agroforestry and forestry systems; some of the cases addressed animal food and medicine being to a greater extent a knowledge associated with men. To a lesser extent, we found knowledge related to human food and medicine in homegardens, even though they are considered women’s space because they are close to the household. Under the lens of FPE and intersectionality, some cases addressed how the gender variable shapes this TAeK, for example, key issues in agriculture and food systems, such as access to seeds, water, land, forests, and labour inputs, which extends to the struggle of men and women to maintain ecologically viable livelihoods, and how race, culture, and ethnicity often interact and shape knowledge construction processes in specific agroecosystems.

However, more work is needed to address the FPE perspective and intersectionality in depth along the lines of agri-food system and TAeK addressed in this systematic review.

In summary, TAeK and agri-food system activities in the different agroecosystems are structured by the gendered division of labour and power relations. These power structures allow women and men to have certain or specific experiences, perceptions, skills, and knowledge of specific activities within the food system, related to production, conservation, and transformation. This gender division of labour and power structures affects men and women in different ways; on the one hand, in some cases it shows that women remain with this TAeK for much longer than men, the women have more often the possibility of transmitting it generationally within the domestic and agroecological spaces, and women play an important role in the economy within the production and marketing spaces. On the other hand, and in certain cases due to the forces of globalization, migration, wide market exposure, and formal education, it seems that the erosion of knowledge is also gendered. This situation is linked to the external drivers of change that following feminist standpoint theory [124] situates women as marginalized actors into a favourable position in addressing current challenges, in our case in food system transformation based on agroecological knowledge. However, such process requires that those barriers and power imbalances suffered by women in food systems (access to land, seed, finance) should be challenged.

Gaps in terms of the current literature have been identified, thematically and geographically. There are agroecosystems very few explored by the literature, such as freshwaters and livestock systems, that deserve to be more researched in the future. Moreover, the topic is barely explored in the global north. In general, a few articles explore the different dimensions of gender, including gendered crops and gendered space in all the agroecosystems. Specifically, though seed conservation is widely explored, little information has been found on the gendered knowledge and task of grain, food, and forage conservation; as well as there is a gap of literature about animal food and veterinary gendered knowledge and tasks.

### Limitations of the review

The literature review shows some limitations and gaps to be further researched. Limitations in the development of this work are related to the search, since only scientific texts in Scopus and in English were examined in depth, limiting the typology of texts analysed on the subject, so a further expansion of the grey literature would be interesting. Another limitation arose because of the  lack of capcity to access some articles, in particular three articles dated the 90s. In addition, works related to fisheries management were excluded, due to the focus of this review of analysis on TAeK in agri-food systems, and it could be convenient to expand the analysis of gender TAeK related to aquatic systems/species. Since articles on mycology that did not study traditional management or did not focus on a case study were excluded, it might be interesting to examine it in depth in relation to gender and agri-food systems, i.e. as a keyword.

## Supplementary Information

**Additional file 1**. Additional methodological information.

## Data Availability

The “Additional file 1” contains the information related to a supplement of information for the Materials and Methods section.  The database in Excel used and/or analysed during the current systematic review study are available from the corresponding author on reasonable request.

## Abbreviations

FPE: Feminist political ecology
ILK: Indigenous local knowledge
IK: Indigenous knowledge
IPBES: Intergovernmental Platform on Biodiversity and Ecosystem Services
IPCC: Intergovernmental Panel on Climate Change
SRCCL: Special Report on Land and Climate Change
TAeK: Traditional agroecological knowledge
SDGs: UN Sustainable Development Goals
WEPs: Wild edible plants

## Footnotes

1 FPE brings feminist theory and objectives to political ecology and suggests gender in relation to class, race, and other relevant axes of power shape access to and control over natural resources and doing so helps to demonstrate how social identities are constituted in and through relationships with nature and everyday material practices [25].

2 Agri-food system is considered a complex system including a series of activities, actors, and interactions along the agri-food value chain from access to resources, input supply, and production of crops, livestock, and other agricultural commodities to transportation, processing, retailing, wholesaling, and preparation of foods to consumption and disposal, contributing to the satisfaction of human food security [27]. Agri-food systems also include the enabling policy environments and cultural norms around food (IFPRI, 2021). In this review, under the lens of the FPE, we also consider access to resources as part of the agri-food system.

3 The agroecosystems concept is adopted from the agroecological studies, where it is conceived as an open system that constantly interacts with the physic, biotic, social, economic, and cultural environment. The agroecosystem is modified by human beings to obtain goods or services with different purposes, and there are also dynamics and relationships between the culture and its physical–biological environment. This concept allows us to cover more than the agroecosystem understood as the agricultural system and incorporate in this analysis surrounding ecosystems that are part of these interactions, such as cropping system, homegarden, agroforestry, forestry, pastoral and silvopastoral, and internal watersheds and wetlands [28].
