# Supplementary material for: Gendered traditional agroecological knowledge in agri-food systems: a systematic review
Source: J Ethnobiol Ethnomed. 2023 Apr 6;19:11. doi: 10.1186/s13002-023-00576-6 (PMC10080974; doi:10.1186/s13002-023-00576-6)
Supplement: Supplementary file 1 — Additional file 1. Additional methodological information. [file 13002_2023_576_MOESM1_ESM.docx]

**Additional file 1. Supplementary information on methods adopted for the systematic review.**

The key words used in relation to the agrifood system, knowledge and gender are shown in Table 1.

Additional file. Table 1 Key words and word association chosen for the research

| **Root key words** | **Additional keywords** |
| --- | --- |
| **Agri-food system** | Agr*/ Agrobiodiversity / Agro-biodiversity / Farm*/ Food/ Agrifood / Agri-food / Agrofood / Agro-food/ Livestock/ Pastor*/ Plant/ Forest |
| **Knowledge** | Local knowledge/ Indigenous knowledge/ Ecological knowledge/ Traditional knowledge/ Folk knowledge/ Cultural/biocultural knowledge |
| **Gender** | Wom*  Fem*  Gender |

A database was created with information from the papers that provided empirical evidence of relationships between the agri-food system, TAeK and gender. In this database we incorporated nine categories of information that are described in table 2.

Additional file. Table 2. Categories of analysis and information integrated in the final database

| **Categories** | **Information** |
| --- | --- |
| Setting | study area, country, continent, type of agroecosystem, practices of use/management, cultural setting, minority/marginalized groups. |
| Focus | Specification of the focus, research areas. |
| Analysis of TAeK | definition of TAeK, scale of the analysis, practice. |
| Identification of TAeK in food system activities | in production, conservation, transformation, and preparation |
|  | for production, specification in gendered crops. |
|  | for conservation specification in grains, seeds, food, or forage suggested a gendered relation. |
|  | for transformation, specification of human food, animal food, veterinary |
| Relation with society | transmission of TAeK, institutions (formal , informal), erosion of TAeK |
| Specific aspects of TAeK and its gendered dimension | on botanical species (e.g., cultivate, uses, management) |
|  | on animal species (e.g., biodiversity, breed, management) |
|  | ecosystem management (e.g., soil, grassland, agroforestry, water management) |
| Drivers of change and adaptation | Indirect drivers: agri-food policies, demographic changes, cultural changes etc.  Direct drivers: environmental changes (climate change; invasive species, etc.).  Gendered impacts  Adaptation measures (gendered) |
| Gender analysis | Feminist approach /gender perspective /women issue  Intersectionality = intersection of gender with other sources of oppression |
| Gendered dimensions of TAeK | Gender access to resources  Gendered institutions (including networks)  Gendered dimensions related to the knowledge, crops, space, tasks, and activities linked to the food system (for each activity) |

The list of papers analyzed during the current study are available from the corresponding author on reasonable request.
